# The Fellowship

**Published:** 1907-09-07

**Authors:** 


### THE FELLOWSHIP.

The Fellowship of the Royal College of Surgeons, justly esteemed as the premier qualification that the student can obtain in surgery, holds out certain attractions which the student would do well carefully to weigh. To obtain it it is necessary to pass two examinations, a preliminary and a final.

The Preliminary examination takes place twice every year, in May and in November, and is held in London. The subjects are Anatomy and Physiology, and candidates must have passed the second Conjoint examination or some other equivalent examination which exempts them from it. They must also (except in the case of qualified men) have spent two winter sessions in dissections and attendance at physiology classes at a recognised school. The examination is partly oral and partly written. Two papers are set, one in physiology and one in anatomy, four questions being given with an allowance of three hours for each paper. The scope of the questions can be easily seen by studying the papers set during the past few years, copies of which may be obtained on application to the Secretary, Examination Hall, Embankment. The oral examinations are two, lasting fifteen minutes each, in the two subjects. To a very large extent the questions asked at the vivas are objective, "spotting," and straightforward questions on regional anatomy, embryology, special anatomy, physiology and histology being usually in the programme. The examination has the reputation of being unusually stiff, with more than the average element of luck appertaining to it, but its difficulty need not frighten the average student or practitioner who is willing to pay special attention to the two subjects. A good, sound knowledge of anatomy and physiology is required, and if the candidate has been diligent at his practical work in the dissecting room and laboratory, and has supplemented the knowledge there gained by reading, he stands a good chance of success.

The examination for the final fellowship takes place in May and November of each year, and consists of written papers, *viva voces*, clinicals, and operative surgery. The fee for the examination is £15 15s., and candidates are required to be members of the college who have been six years in practice or the study of medicine.

